# The Induction of Oxalate Metabolism *In Vivo* Is More Effective with Functional Microbial Communities than with Functional Microbial Species

**DOI:** 10.1128/mSystems.00088-17

**Published:** 2017-09-26

**Authors:** Aaron W. Miller, Colin Dale, M. Denise Dearing

**Affiliations:** a Departments of Urology and Immunology, Cleveland Clinic, Cleveland, Ohio, USA; b Department of Biology, University of Utah, Salt Lake City, Utah, USA; Dalhousie University

**Keywords:** fecal transplant, gut microbiota, oxalate, probiotics, woodrat

## Abstract

For mammals, oxalate enters the body through the diet or is endogenously produced by the liver; it is removed by microbial oxalate metabolism in the gut and/or excretion in feces or urine. Deficiencies in any one of the these pathways can lead to complications, such as calcium oxalate urinary stones. While considerable research has been conducted on individual oxalate-degrading bacterial isolates, interactions between oxalate and the gut microbiota as a whole are unknown. We examined the reduction in oxalate excretion in a rat model following oral administration of fecal microbes from a mammalian herbivore adapted to a high oxalate diet or to fecal transplants consisting of two different formulations of mixed oxalate-degrading isolates. While all transplants elicited a significant reduction in oxalate excretion initially, the greatest effect was seen with fecal microbial transplants, which persisted even in the absence of dietary oxalate. The reduction in oxalate excretion in animals given fecal transplants corresponded with the establishment of diverse bacteria, including known oxalate-degrading bacteria and a cohesive network of bacteria centered on oxalate-degrading specialists from the *Oxalobacteraceae* family. Results suggested that the administration of a complete community of bacteria facilitates a cohesive balance in terms of microbial interactions. Our work offers important insights into the development of targeted bacteriotherapies intended to reduce urinary oxalate excretion in patients at risk for recurrent calcium oxalate stones as well as bacteriotherapies targeting other toxins for elimination.

**IMPORTANCE** Oxalate is a central component in 80% of kidney stones. While mammals do not possess the enzymes to degrade oxalate, many gastrointestinal bacteria are efficient oxalate degraders. We examined the role of cohesive microbial networks for oxalate metabolism, using Sprague-Dawley rats as a model host. While the transplantation of oxalate-degrading bacteria alone to the Sprague-Dawley hosts did increase oxalate metabolism, fecal transplants from a wild mammalian herbivore, *Neotoma albigula*, had a significantly greater effect. Furthermore, the boost for oxalate metabolism persisted only in animals that received fecal transplants. Animals receiving fecal transplants had a more diverse and cohesive network of bacteria associated with the *Oxalobacteraceae*, a family known to consist of specialist oxalate-degrading bacteria, than did animals that received oxalate-degrading bacteria alone. Our results indicate that fecal transplants are more effective at transferring specific functions than are microbial specialists alone, which has broad implications for the development of bacteriotherapies.

## Abstract (precis)

Oxalate is a central component in 80% of kidney stones. While mammals do not possess the enzymes to degrade oxalate, many gastrointestinal bacteria are efficient oxalate degraders. We examined the role of cohesive microbial networks for oxalate metabolism, using Sprague-Dawley rats as a model host. While the transplantation of oxalate-degrading bacteria alone to the Sprague-Dawley hosts did increase oxalate metabolism, fecal transplants from a wild mammalian herbivore, *Neotoma albigula*, had a significantly greater effect. Furthermore, the boost for oxalate metabolism persisted only in animals that received fecal transplants. Animals receiving fecal transplants had a more diverse and cohesive network of bacteria associated with the *Oxalobacteraceae*, a family known to consist of specialist oxalate-degrading bacteria, than did animals that received oxalate-degrading bacteria alone. Our results indicate that fecal transplants are more effective at transferring specific functions than are microbial specialists alone, which has broad implications for the development of bacteriotherapies.

## INTRODUCTION

At a cost of $10 billion in direct medical expenses annually in the United States, urinary stone disease (USD) is the most expensive urological condition in the United States and has seen a 3-fold increase in prevalence over the last 40 years (1, 2). Approximately 80% of kidney stones consist primarily of calcium oxalate (3). Despite the toxicity of oxalate and the fact that it is produced by endogenous enzymes, mammals do not produce enzymes to degrade it (4, 5); instead, mammals rely on gastrointestinal bacteria for oxalate metabolism (6–14). While considerable research has been conducted on individual oxalate-degrading isolates, interactions between the gut microbiota as a whole and oxalate metabolism have received little attention.

The factors that facilitate the colonization, persistence, and function of oxalate-degrading bacteria within the mammalian gut are unknown. Some mammalian herbivores harbor oxalate-degrading bacteria for generations after being brought into captivity and fed a diet lacking oxalate (15). However, when humans or small mammals are administered oxalate-degrading bacteria orally, these microbes typically persist in the gut for only a few days or weeks before becoming undetectable in the feces (16–18). Additionally, some patients with primary hyperoxaluria administered *Oxalobacter formigenes* twice daily for 8 weeks exhibited substantial enrichment of *O. formigenes* in their stool with no significant reduction in urinary oxalate, which was the intended outcome (19). Thus, in these patients, colonization by an oxalate-degrading specialist bacterium did not appear to induce a change in the oxalate-degrading function.

Certain mammalian herbivores persist naturally on a high oxalate diet and therefore provide an excellent model for the study of interactions between microbes and dietary oxalate. One notable example is the white-throated woodrat (*Neotoma albigula*), which consumes a high-oxalate (~1.5%) diet derived from a diet that is almost exclusively composed of *Opuntia* sp. cactus (20). Consumption of oxalate by these animals stimulates a broad response within the gut microbiota, which degrades ~100% of the oxalate consumed (21–23). These responses are in contrast to those in animals that have a poor capacity for oxalate degradation, in which there is a decrease in microbial diversity and an increase in oxalate excretion in accordance with exposure to oxalate (16–24). When such animals are administered a fecal transplant from *N. albigula*, they exhibit a significant and persistent reduction in oxalate excretion and maintain elevated levels of oxalate-degrading bacteria for up to 9 months (24).

To obtain a deeper understanding of the role of the mammalian microbiota in oxalate metabolism, we compared the effectiveness of transplanting whole fecal microbial communities versus two different groups of mixed oxalate-degrading isolates for the ability to reduce oxalate excretion *in vivo*. Our central objective was to quantify changes in oxalate degradation after microbial transplantation of the cultured oxalate-degrading isolates versus the oxalate-degrading community. Additionally, we tracked changes in the gut microbiota of donor and recipients across variable oxalate diets and microbial transplants and identified microbes that were associated with the family *Oxalobacteraceae*, of which *Oxalobacter* is a member. To accomplish these objectives, we utilized a nonmodel species, *N. albigula*, which harbors a broad diversity of oxalate-degrading bacteria that are stimulated by oxalate consumption (20–23).

## RESULTS

In the current study, we sought to compare the efficiencies of three microbial transplants and controls in facilitating increased oxalate degradation in a model mammalian host, the Sprague-Dawley rat (SDR). These treatments consisted of: (i) VSL#3, a probiotic developed to treat irritable bowel disorders, which also has some capacity to decrease urinary oxalate excretion in humans (25–28); (ii) NA bacteria, which were cultured oxalate-degrading bacteria that were previously isolated from *N. albigula* (20); and (iii) NA feces, that is, the whole fecal microbial community from *N. albigula*. The first two preparations represent cultured subsets of oxalate-degrading bacteria isolated from human and *N. albigula* sources, respectively, while the third treatment represents a mammalian fecal microbiota in its entirety.

### Effect of microbial transplants on oxalate degradation and host physiology.

**FIG 1:**
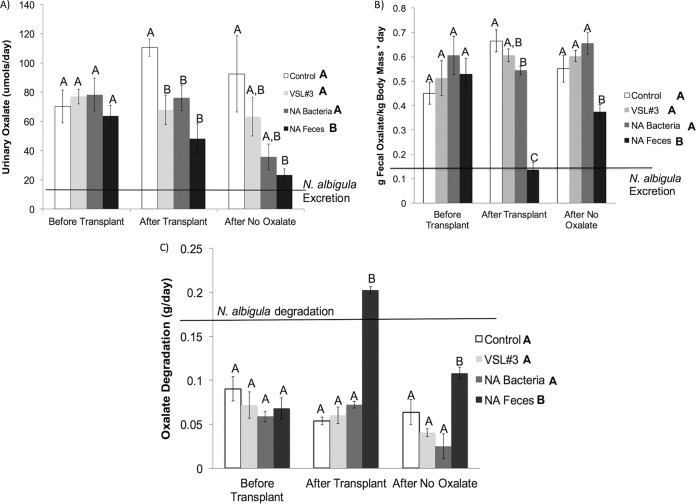
Oxalate metrics over the course of the experiment. Significant treatment differences among groups were determined via a repeated-measures ANOVA, for which the *P* values and degrees of freedom (in parentheses) for effect and error, respectively, were as follows: for oxalate degradation, treatment (3, 18), *P* < 0.001; time (2, 48), *P* < 0.001; treatment × time (8, 66), *P* < 0.001; for urinary oxalate, treatment (3, 18), *P* = 0.002; time (2, 48), *P* < 0.001; treatment × time (8, 66), *P* = 0.693; for fecal oxalate, treatment (3, 18), *P* ≤ 0.001; time (2, 48), *P* < 0.001; treatment × time (8, 66), *P* ≤ 0.001. Treatment differences are indicated by letters in the legend. Letters above columns indicate significant differences between treatments within a time point, as assessed by a *post hoc*, Holm’s-corrected Tukey’s analysis. The black lines indicates *N. albigula* degradation and excretion. Animals were consuming 1.5% oxalate at each of the time points shown.

There were significant differences in some metrics during the transplant period, with the group receiving fecal transplants from *N. albigula* (NA feces group) exhibiting significantly greater food intake and fecal output than the other groups but lower dry matter digestibility (DMD) during this period. No significant differences were found in the slope of the curve for body mass change, oxalate intake, water intake, or urine output (Table 1; see also Fig. S1 in the supplemental material). All microbial transplants initially resulted in reduced urinary oxalate relative to the control group, and all but VSL#3 caused reduced fecal oxalate (Fig. 1A and B). However, neither the NA bacteria nor VSL#3 transplants produced a significant change in total oxalate degradation (Fig. 1C). In contrast, animals treated with NA feces exhibited oxalate degradation above the baseline level for *N. albigula* (Fig. 1C). For the NA feces transplant group, the effects of microbial transplants on oxalate degradation and excretion only persisted after the no-oxalate period (Fig. 1A to C).

10.1128/mSystems.00088-17.1 FIG S1  Significant physiological metrics over the course of the experiment. Treatment differences are indicated by letters in the legend. Letters above columns indicate significant differences between treatments within a time point, as assessed by a *post hoc*, Holm’s-corrected Tukey’s analysis. Animals were consuming 1.5% oxalate at each of the time points shown. (A) Food intake; (B) fecal output; (C) DMD. Download FIG S1, TIF file, 2.1 MB. Copyright © 2017 Miller et al. 2017 Miller et al. This content is distributed under the terms of the Creative Commons Attribution 4.0 International license.

**TABLE 1:** Statistics for host metrics^a^

Metric	Mean ± SE	Treatment			Time			Treatment × time		
		df	F	*P*	df	F	*P*	df	F	*P*
Food intake (g/kg body wt)	64.54 ± 1.18	3, 20	3.01	0.037	2, 15	16.6	<0.001	6, 68	2.83	0.017
Fecal output (g/kg body wt)	12.59 ± 0.71	3, 20	10.70	<0.001	2, 15	5.21	0.002	6, 68	10.97	<0.001
Dry matter digestibility (%)	79.74 ± 0.1	3, 20	2.34	0.082	2, 15	0.06	0.95	6, 68	3.97	0.002
Slope of body mass change	0.007 ± 0.0003	3, 20	1.95	0.150	NA	NA	NA	NA	NA	NA
Oxalate intake (g/kg body wt)	0.904 ± 0.009	3, 20	1.44	0.240	2, 15	2.58	0.06	6, 68	0.64	0.69
Water intake (ml)	21.4 ± 0.47	3, 20	2.56	0.060	2, 15	2.46	0.09	6, 68	1.18	0.33
Urine output (ml)	5.68 ± 0.2	3, 20	2.26	0.090	2, 15	2.71	0.07	6, 68	0.19	0.98

a Global means were determined after pooling data for all SDR animals across all time points. Data for each treatment and time point across the whole experiment were analyzed with a repeated-measures ANOVA. NA, not applicable.

### Effects of microbial transplants on the gut microbiota.

Responses of the gut microbiota to microbial transplants and oxalate exposure were determined through high-throughput sequencing of the 16S rRNA gene. Sequencing efforts resulted in 7,395,662 high-quality sequences with 32,927 unique operational taxonomic units (OTUs) defined. The communities of all six animals in each group were represented by a high abundance (>20,000) of reads for each time point. A total of 95.3% of all OTUs were assigned at the phylum level and 47.2% to the level of genus. While *N. albigula* was dominated by *Bacteroides* (55%), SDR were dominated by *Firmicutes* (46%), with the exception of the NA feces group after the transplant, which was then dominated by *Bacteroides* (50% of the community).

**FIG 2:**
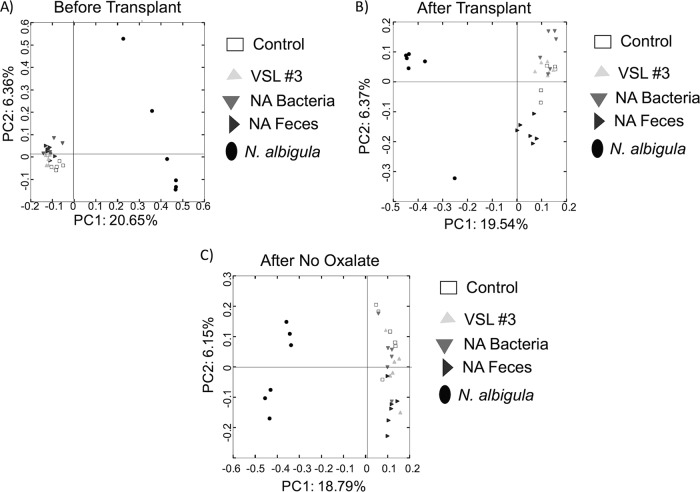
PCoA plots of the β-diversity (unweighted UniFrac analysis) by time point. Statistical differences between SDR treatment groups across the whole trial were determined by using ANOSIM (*P* = 0.001, *R* = 0.39). Animals consumed 1.5% oxalate at each of the time points shown.

**FIG 3:**
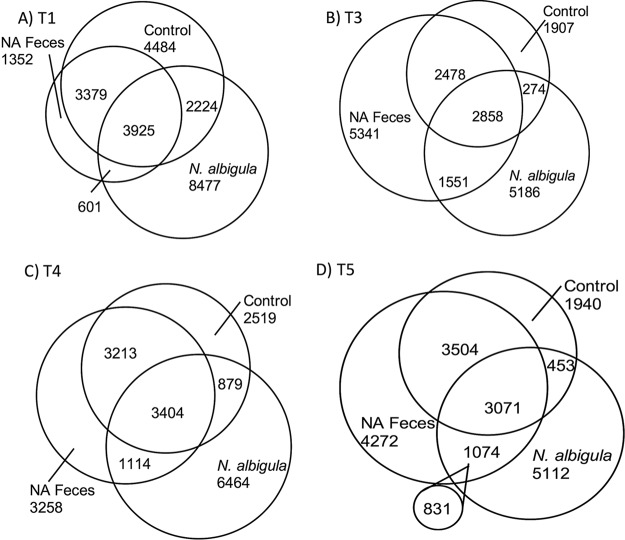
**γ-**Diversity, reflecting the total number of overlapping and nonoverlapping OTUs between the control, NA feces, and *N. albigula* treatment groups. Time points: T1, before transplant (1.5% oxalate); T3, after transplant (1.5% oxalate); T4, no-oxalate period (0% oxalate), T5, after no-oxalate period (1.5% oxalate). Circles represent specific groups, and numbers reflect the number of OTUs. In panel D, the small circle outside the overlapping circles contains the numbers of taxa shared between *N. albigula* and the NA feces treatment group at T5 but not at T3.

**FIG 4:**
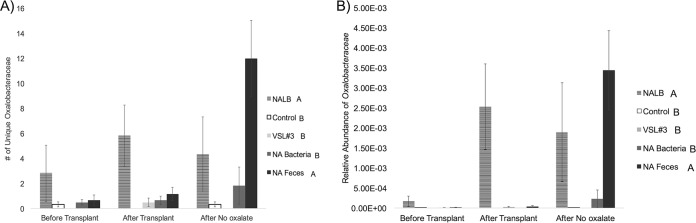
*Oxalobacteraceae* found in each group before transplant, after transplant, and after no oxalate exposure. Significance between groups was determined with a repeated-measures ANOVA. Letters next to groups indicate significantly different treatments across the experiment as assessed by a *post hoc*, Holm’s-corrected Tukey’s analysis. For each panel, the degrees of freedom (in parentheses) for effect and error, respectively, were as follows. (A) Number of unique *Oxalobacteraceae*. Treatment (4, 24), *P* < 0.001; time (2, 60), *P* = 0.008; treatment × time (14, 84), *P* = 0.002. (B) Relative abundance of *Oxalobacteraceae*. Treatment (4, 24), *P* < 0.001; time (2, 60), *P* = 0.006; treatment × time (14, 84), *P* = 0.002. Animals were consuming 1.5% oxalate at each of the time points shown.

Across the entire experiment, the composition of the microbiota from SDR animals clustered by microbial transplant treatment, as assessed by principle coordinates analysis (PCoA) (Fig. 2) and a *post hoc* analysis of similarity (ANOSIM; *R* = 0.39, *P* = 0.001). Before microbial transplants, all microbiota from SDR animals clustered together but separately from *N. albigula*. *Post hoc*, false-discovery rate (FDR)-corrected pairwise comparisons of the unweighted UniFrac distances between each microbial transplant group of the SDR animals, directly after the transplant period and at the end of the experiment, revealed that the NA bacteria and NA feces groups exhibited significant clustering away from the control group during these time points, while the VSL#3 transplant group showed no effect (Fig. 2; Table 2). The γ-diversity for the control, NA feces, and *N. albigula* groups revealed considerable overlap in the native microbiota of *N. albigula* and SDR (Fig. 3). Additionally, VSL#3 and NA bacteria transplant groups initially inflated the γ-diversity of the gut microbiota (Fig. S2 and S3). While many OTUs were transferred to the NA feces group via fecal transplants, there was considerable turnover from the initial transfer to the end of the trial, whereby 831 of the 1,074 OTUs unique to both *N. albigula* and the NA feces group at the end of the trial were not present immediately after the transfer (Fig. 3). This included the emergence of a diversity of *Oxalobacteraceae*, few of which were detected immediately after the transplant. The increase in *Oxalobacteraceae* diversity corresponded to an increase in *Oxalobacteraceae* relative abundance (Fig. 4).

10.1128/mSystems.00088-17.2 FIG S2  Gamma diversity, reflecting the total number overlapping and nonoverlapping OTUs between control and VSL#3 groups. Time points: T1, before transplant; T3, after transplant; T4, no-oxalate period; T5, after no-oxalate period. Circles represent specific groups and numbers reflect the number of OTUs. Animals were consuming 1.5% oxalate at each of the time points shown. Download FIG S2, TIF file, 1.1 MB. Copyright © 2017 Miller et al. 2017 Miller et al. This content is distributed under the terms of the Creative Commons Attribution 4.0 International license.

10.1128/mSystems.00088-17.3 FIG S3  Gamma diversity, reflecting the total number overlapping and nonoverlapping OTUs between control and NA bacteria groups. Time points: T1, before transplant; T3, after transplant; T4, no-oxalate period; T5, after no-oxalate period. Circles represent specific groups and numbers reflect the number of OTUs. Animals were consuming 1.5% oxalate at each of the time points shown. Download FIG S3, TIF file, 1.1 MB. Copyright © 2017 Miller et al. 2017 Miller et al. This content is distributed under the terms of the Creative Commons Attribution 4.0 International license.

**TABLE 2:** Pairwise ANOSIM results for the unweighted UniFrac distance matrices

Group 1	Group 2	*P* value (after/end)^a^
Control	WR bacteria	0.02/0.05
Control	VSL#3	0.65/0.91
Control	WR feces	0.03/0.02
VSL#3	WR bacteria	0.05/0.08
VSL#3	WR feces	0.02/0.02
WR bacteria	WR feces	0.03/0.02

a We compared the microbial composition of SDR fecal communities from immediately “after” the transplant period and at the “end” of the diet trial. *P* values were FDR corrected for multiple comparisons. WR, whole rat.

**FIG 5:**
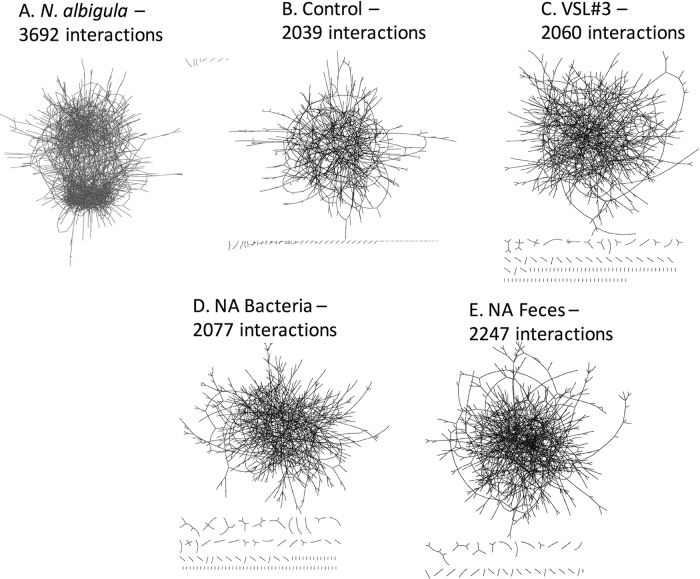
Multilayered cooccurrence network analysis of OTUs that exhibited a significant positive Spearman’s correlation with oxalate consumption at the end of the diet trial. Nodes, which have been minimized to enable clear viewing of entire networks, represent individual OTUs, and edges represent a significant interaction between two OTUs. Dashes represent isolated interactions of a few OTUs. Animals were consuming 1.5% oxalate at the time of sampling.

Across the data set, approximately 7% (2,378 out of 32,927) of the OTUs exhibited a significant positive correlation with *Oxalobacteraceae*, bacteria that include the oxalate specialist *Oxalobacter*, while 4 OTUs exhibited a significant negative correlation (Table S1). The OTUs that were significantly correlated with *Oxalobacteraceae* were dominated by the S24-7 family, followed by OTUs from the *Clostridiales* order. For network analysis, only those OTUs exhibiting a significant positive correlation were used. The number of significant positive interactions, as assessed with SparCC, between treatment groups varied from 2,039 to 3,692 at the last time point (Fig. 5). The *N. albigula* group harbored the most extensive number of interactions with the fewest number of isolated interactions. Of the microbial transplant groups, the NA feces treatment group had the most interactions and fewest isolated instances, while all other groups had similar levels of total and isolated interactions. The list of bacteria exhibiting a significant correlation with *Oxalobacteraceae* was compared to the list of bacteria exhibiting a significant correlation with oxalate consumption in a previous study (A. W. Miller, C. Dale, and M. D. Dearing, submitted for publication). Approximately 10% of the OTUs that correlated with oxalate consumption in the Miller et al. study also correlated with *Oxalobacteraceae* in the current study. However, at the family level, 82% of the taxa that correlated with oxalate consumption also correlated with *Oxalobacteraceae* in this study.

10.1128/mSystems.00088-17.4 TABLE S1  Microbial OTUs (out of 32,927) that exhibited a positive significant Pearson’s correlation with the relative abundance of *Oxalobacteraceae*. Download TABLE S1, PDF file, 0.4 MB. Copyright © 2017 Miller et al. 2017 Miller et al. This content is distributed under the terms of the Creative Commons Attribution 4.0 International license.

## DISCUSSION

Numerous attempts have been made to develop probiotics that effectively reduce urinary oxalate excretion. While probiotics containing *O. formigenes*, *Lactobacillus* spp., *Bifidobacterium* spp*.*, or *Enterococcus* spp*.* effectively reduce urinary oxalate excretion, the bacteria and the oxalate-degrading function are often lost when probiotic use ceases and/or oxalate is removed from the diet (10, 16, 18, 19, 27–31). In some cases, urinary oxalate remains unchanged despite long-term administration of probiotics and persistence of oxalate-degrading microbes in the gut (19). Thus, there is a need to understand the conditions that facilitate persistent and effective microbial oxalate metabolism in the gut (30, 31). In the current study, the NA feces group exhibited a 66% reduction in urinary oxalate after 3 days of consecutive transplants, which is on par with results of studies that administered daily oral doses of *O. formigenes* for up to 4 weeks (16–18). Furthermore, in contrast to previous studies using a single strain of *O. formigenes* as a probiotic, the diversity and relative abundance of the *Oxalobacteraceae* increased considerably over time to a level that was greater than in the donors, even after a week on a diet lacking oxalate (Fig. 4). Similar results were obtained in previous fecal microbial transplants from *N. albigula*, whereby the *Oxalobacteraceae* and the oxalate-degrading function persisted for 9 months following transplants, even though the animals were maintained on a low-oxalate diet (24).

The differences observed in persistence of oxalate-degrading bacteria in the treatment groups in this study can be explained in a number of ways. First, and perhaps most likely, there may be a requirement for a cohesive microbial network that includes both oxalate-degrading bacteria along with microbes that provide supportive metabolic functions. Although the VSL#3 and NA bacteria transplants contained an order of magnitude greater number of oxalate-degrading bacteria than the NA feces transplants, these groups had a transient and reduced effect on oxalate degradation/excretion compared to the NA feces whole-community transplants. Both the VSL#3 and NA bacteria transplants were mixtures of bacteria obtained from laboratory culture. Culturing is known to introduce a significant bias toward organisms that grow well in the artificial environment and can therefore exclude bacteria that perform important functions within microbial communities in the gut environment (32). Interestingly, the NA feces transplants resulted in the transfer of a far greater number of OTUs that exhibited a significant correlation with the *Oxalobacteraceae* than the VSL#3 and NA bacteria transplants, with greater cohesiveness in the resulting microbial network. The microbial network associated with *Oxalobacteraceae* was particularly dominated by the S24-7 family. Recent genomic analysis has shown that this family harbors a complete oxalate metabolic pathway within their genome (33). Thus, the correlation between the S24-7 and *Oxalobacteraceae* may in part be driven by the common function of oxalate degradation. *Ralstonia*, a genus of the *Oxalobacteraceae* family, exhibited the greatest level of correlation. Like its congeneric *Oxalobacter*, *Ralstonia* is an oxalate-degrading genus (34). The γ-diversity analysis revealed that there were ~1,000 OTUs unique to both the microbiota of *N. albigula* and the NA feces transplant group that were not present in the controls. It is the bacteria within this group or other undetectable bacteria that must drive the differences in oxalate metabolism between the different treatments, as oxalate metabolism is exclusively a microbial function.

Another factor that may impact the persistence of bacteria associated with oxalate metabolism is the presence of fecal material within the oral preparation. Given that woodrats and many other rodents are naturally coprophagic (35), one would assume that their microbiota is adapted to colonization of the gastrointestinal tract through the reingestion of feces. In contrast, bacteria that are obtained from culture and administered in a purified form might not express the genes necessary for colonization and/or they might not be able to survive the transition to the gastrointestinal tract. The difference observed between the VSL#3 and NA bacteria transplants may also reflect the fact that VSL#3 microbes are of human origin, whereas the NA bacteria group organisms were isolated from rodents. Success of microbial transplantation is anticipated to be higher when the evolutionary distance between the donor and recipient host is smaller (36). Regardless of the mechanisms facilitating persistence, our study contributes additional evidence that whole-community transplants are more effective and persistent than those with isolated probiotics (37–40).

The reduction in oxalate excretion after microbial transplant may be due to several potential factors. First, microbial transplants may mitigate the negative effects of oxalate on the microbiota. The persistent consumption of oxalate can gradually increase oxalate excretion over time in animals that exhibit poor levels of oxalate degradation initially, even when dietary sources remain constant, and this is indicative of a toxic effect of oxalate for the microbiota (16–24) (Fig. 1). Second, transplants may increase total oxalate degradation within the gut (24, 41) (Fig. 1). With increased oxalate degradation in the gut, the amount of oxalate available for excretion is reduced. Finally, the transplantation of certain bacteria may induce the secretion of oxalate from the blood into the lower gastrointestinal tract, where it is exposed to the gut microbiota. This effect is known to occur with *O. formigenes* (42). However, a recent clinical trial with primary hyperoxaluria patients, who are genetically predisposed to excess endogenous oxalate production, involved the administration of *O. formigenes* twice daily for 8 weeks. Over the course of the trial, urinary oxalate excretion did not differ between patients receiving *O. formigenes* and those receiving a placebo, despite the treatment group having substantial levels of the bacteria at the end of the trial, which suggests that oxalate secretion was not induced (19). Regardless, the potential mechanisms involved in the reduction of oxalate after microbial transplants are complex, and evidence suggests that some combination of all of them are at play with different types of transplants.

In the current study, all microbial transplants were well tolerated, with no apparent changes in health, behavior, or stool consistency. However, fecal transplants did appear to have a temporary effect on digestibility, as indicated by an increase in food intake and fecal output, along with a decrease in DMD. These effects had subsided by the end of the diet trial.

**FIG 6:**
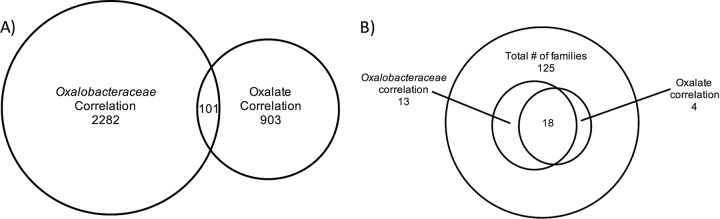
A comparison of techniques to identify the bacteria associated with oxalate metabolism, correlating specific OTUs to either oxalate consumption (black) or to cooccurrence with *Oxalobacteraceae* (gray). The shared taxa between the two techniques are shown at the level of OTU (A) and family (B).

The study presented here offers several significant advances in the development of oxalate-degrading probiotics. We have shown that oxalate-adapted whole microbial communities are significantly more effective than microbial transplants that are comprised of oxalate-degrading species alone. In other words, our results show that the use of a few bacteria with specific functions does not necessarily yield an effective and persistent probiotic. Following fecal transplants, the diversity and relative abundance of the oxalate-degrading *Oxalobacteraceae* increased significantly over time, in stark contrast to previous studies with isolates. Additionally, we were able to elucidate a cohesive microbial network associated with the *Oxalobacteraceae* family. The overlap in the composition of the microbial network associated with *Oxalobacteraceae* and associated with oxalate consumption (Fig. 6) (Miller et al., submitted) suggests that these results represent a consistent, cohesive, and biologically meaningful network. If an analogous network can be identified in humans, it will likely prove useful in the diagnosis and treatment of USD. While the woodrat microflora cannot presently be considered a viable treatment for USD, it is anticipated to yield further useful insight into the development of probiotics and synbiotics capable of inducing a persistent network of oxalate-degrading bacteria in the gut microbiota of patients at risk for recurrent calcium oxalate stones.

## MATERIALS AND METHODS

### Oxalate diet trials and microbial transplants.

Experiments consisted of five treatment groups with 6 animals each for a total of 30 animals. *N. albigula* rats were collected from Castle Valley, UT (38.63°N, 109.41°W) in September 2014 by using Sherman live traps. Animals were transported to the animal facility at the University of Utah and maintained in individual cages (48 by 27 by 20 cm) and fed a 0.2% oxalate, high-fiber rabbit chow diet (Harlan Teklad formula 2031) for 30 days at 28°C and 20% humidity prior to experimentation. These animals were used as a positive controls and fecal donors. For all other treatment groups, 24 female SDR were purchased (20 to 21 days old) from Harlan Laboratories (Denver, CO). The SDR were cohoused five to a cage and fed standard rat chow for 45 days prior to experimentation. All methods were approved by the IACUC under protocol 12-12010.

Microbial transplant groups received one of the following: (i) VSL#3, a probiotic developed to treat irritable bowel disorders and which also has some capacity to decrease urinary oxalate excretion in humans (25–28); (ii) NA bacteria, which are cultured oxalate-degrading bacteria that were previously isolated from the white-throated woodrat, *N. albigula* (20); (iii) NA feces, i.e., the whole fecal microbial community from *N. albigula*. The first two preparations represent cultured subsets of oxalate-degrading bacteria isolated from human and *N. albigula* sources, respectively (Table 3), while the third treatment represents a mammalian fecal microbiota in its entirety. Fecal transplants were prepared by grinding 1 g of fresh or autoclaved feces per day for each recipient from *N. albigula* donors with a sterile mortar and pestle and adding it directly to the food. The negative-control group received autoclaved feces, while the NA feces group received fresh feces. Positive controls included animals that received the *N. albigula* microbiota in its native host. The degradation of dietary oxalate was monitored by subtracting the total amount of oxalate excreted from the amount of oxalate consumed (23, 24).

**TABLE 3:** Bacteria present in the NA bacteria or VSL#3 group

NA bacteria	VSL#3
*Lactobacillus reuteri*	*Bifidobacterium breve*
*L. animalis*	*B. longum*
*L. johnsonii*	*B. infantum*
*L. gasseri*	*L. paracasei*
*Enterococcus gallinarum*	*L. plantarum*
	*L. bulgaris*
	*Streptococcus thermophilus*

The VSL#3 and NA bacteria preparations were included to provide oxalate-degrading bacteria without the additional bacteria that may be a part of an overall microbial network associated with oxalate metabolism. For VSL#3, freeze-dried preparations were added to sterile deionized water, vortexed, centrifuged, and decanted. For NA bacteria, an overnight culture of each species of bacteria was grown and mixed in equal proportions by volume. The community was then centrifuged and supernatant was decanted. From the pelleted bacteria of each preparation, 38 mg was added directly to the food, which was chosen based on previous studies that used *O. formigenes* probiotics for rodents (18). Preparations corresponded to approximately 5 × 10^8^ oxalate-degrading bacteria for the VSL#3 preparation and 7 × 10^8^ oxalate-degrading bacteria for the NA bacteria group. The number of oxalate-degrading bacteria in VSL#3 and NA bacteria preparations were comparable to the total number of potential oxalate-degrading bacteria in the NA feces group, 2 × 10^7^. Quantification of bacterial numbers was performed through absorbance of the microbial preparations on a spectrophotometer at 600 nm, calibrated to direct microscopic counts. For fecal transplants, the total bacteria count was multiplied by the relative abundance for all potential oxalate-degrading bacteria in microbial inventories, as described by Miller et al. (20, 24).

All animals received the same dietary regimen (Table S2). During the diet trial, a custom rat chow with 0% oxalate was used (Table S3). For *N. albigula*, the custom rat chow was mixed in a 3:1 ratio with high-fiber rabbit chow so that animals would consume the diet and maintain body mass. The mix increased the baseline oxalate levels of the custom diet to 0.05%. To make 1.5% (weight/weight) oxalate diets, sodium oxalate (Fisher Scientific, Pittsburgh, PA) was added to the diet. The first no-oxalate period allowed for the quantification of the excretion of endogenous oxalate. The first 1.5% oxalate period allowed for the quantification of dietary oxalate excretion and degradation in animals with their native microbiota and to acclimate the *N. albigula* microbiota to oxalate degradation. Microbial transplants were given with oxalate both to facilitate colonization by oxalate-degrading bacteria and to quantify the change in oxalate degradation. The second no-oxalate period was intended to remove transient oxalate-degrading bacteria. The final oxalate period was intended to quantify the persistence of colonized bacteria and the oxalate-degrading function.

10.1128/mSystems.00088-17.5 TABLE S2  Timeline for the diet trial to quantify the effect of oxalate and microbial transplants on oxalate degradation (feces were collected for microbial inventories at the end of each time point). Download TABLE S2, PDF file, 0.1 MB. Copyright © 2017 Miller et al. 2017 Miller et al. This content is distributed under the terms of the Creative Commons Attribution 4.0 International license.

10.1128/mSystems.00088-17.6 TABLE S3  Ingredients of the purified rat chow diet. Download TABLE S3, PDF file, 0.02 MB. Copyright © 2017 Miller et al. 2017 Miller et al. This content is distributed under the terms of the Creative Commons Attribution 4.0 International license.

Animals were placed in metabolic cages for the duration of the diet trial to separate urine and feces and to allow quantification of food and water intake, which was given *ad libitum*. Urine and feces were collected daily for oxalate assays, and feces were also used for microbial inventories at the end of each time period. Prior to oxalate assays, urine was frozen (−20°C) and feces were dried at 45°C overnight. For microbial inventories, a portion of fecal samples was frozen at −80°C.

Oxalate excretion was quantified with KMnO_4_ titration as previously described (23, 24). Briefly, acidified urine was thawed and centrifuged to remove precipitates. The pH was brought up to 7 with 3 M NaOH, followed by the addition of 0.1 g of CaCl_2_ to precipitate calcium oxalate. Urine was then centrifuged and decanted to collect calcium oxalate. A volume of deionized water equal to the starting volume of urine was added to calcium oxalate precipitate, and the solution was titrated as described below. For fecal oxalate extraction, dried fecal samples were ground with a mortar and pestle and acidified with 6 N H_2_SO_4_ for 15 min to extract oxalate. Precipitates were filtered from samples with a grade 4 Whatman filter, and the pH of the filtrate was raised to 7 with NaOH. Calcium oxalate was precipitated with CaCl_2_ and isolated by centrifugation. A volume of deionized water equal to the volume of the filtrate was added prior to titration. Calcium oxalate solutions were acidified and heated to 80°C prior to titration with 0.01 M KMnO_4_. Titration volumes were then compared to standards with known amounts of oxalate added. These methods allowed for the recovery of 90 to 110% of urinary and fecal oxalate (23, 24). Oxalate degradation was conservatively estimated as the oxalate consumed minus the oxalate excreted. We also quantified body mass, DMD, oxalate consumption, and oxalate degradation. Data were analyzed with a repeated-measures analysis of variance (ANOVA) and a *post hoc* Tukey’s analysis.

### Microbial inventories.

To track fecal microbial communities, DNA was extracted from 180 to 220 mg of feces with the QIAamp DNA stool minikit (Qiagen, Germantown, MD). A total of 120 inventories were generated from all animals at all time points specified in Table S4, except for the first 1.5% oxalate period before the treatments. This time point was excluded because there were no differences in oxalate degradation among the SDR groups. Fecal DNA was sent to Argonne National Laboratory (Chicago, IL) for sequencing of the V4 region of the 16S rRNA gene, using primers 515F and 806R (43). This gene is typically used in the identification of bacterial OTUs (44) and, when combined with high-throughput sequencing, results in an inventory of OTUs and their relative abundances (45). Barcodes with 12 bp were added to the amplified gene region and samples were multiplexed on a single-lane, MiSeq run with 150 paired-end sequencing (43).

10.1128/mSystems.00088-17.7 TABLE S4  List of samples sequenced for 16S rRNA inventories. Download TABLE S4, PDF file, 0.02 MB. Copyright © 2017 Miller et al. 2017 Miller et al. This content is distributed under the terms of the Creative Commons Attribution 4.0 International license.

Sequencing data were demultiplexed and quality control included use of default parameters in QIIME (45). OTUs were assigned *de novo* with UCLUST at a cutoff of 97% sequence similarity. Sequences corresponding to chloroplasts or mitochondria or that had fewer than 10 representations across the whole data set were removed. A negative binomial Wald test, via the DESEQ2 algorithm, was used to normalize data across samples to maintain rare taxa (46, 47).

With the normalized OTU table, we quantified β-diversity with unweighted and weighted UniFrac analyses to examine community membership and structure, respectively. For statistical comparison, we performed an ANOSIM for microbial transplant groups, excluding the microbiota from the *N. albigula* animals, within each time point after 99 permutations. If significant clustering was present among the microbiota from the different groups, we conducted a *post hoc* pairwise analysis with an FDR correction. We also quantified γ-diversity among relevant groups as the total number of shared or unique OTUs in each group. The average numbers of unique OTUs in the microbiota for animals in each treatment group were compared with a repeated-measures ANOVA and *post hoc* pairwise *t* tests with FDR correction of the *P* values, in order to examine the effect of time and transplants on the microbiota.

Quantification of the microbial network associated with oxalate metabolism was based on the assumption that bacteria in the *Oxalobacteraceae* family, which utilize oxalate as a carbon and energy source, are central to the network and respond with other OTUs within the network. Therefore, FDR-corrected, repeated-measures Pearson correlations were performed between OTU relative abundance levels and the relative abundance of all OTUs in the *Oxalobacteraceae* family for all samples, using the R statistical package. A list restricted to the bacteria that exhibited a significant positive correlation with *Oxalobacteraceae* was used for downstream network analysis. From the restricted list, a SparCC cooccurrence analysis was performed for all groups at the last time point of the diet trial (48). Only cooccurrences with a *P* value of 0 were used for network visualization. Cooccurrence networks were visualized in Cytoscape (49).

### Accession number(s).

The accession number assigned for the high-throughput sequence reads, which were submitted to the Sequence Read Archive, is SRR5261472.
