# Supplementary figures and images for: The Induction of Oxalate Metabolism In Vivo Is More Effective with Functional Microbial Communities than with Functional Microbial Species
Source: mSystems. 2017 Sep 26;2(5):e00088-17. doi: 10.1128/mSystems.00088-17 (PMC5613171; doi:10.1128/mSystems.00088-17)

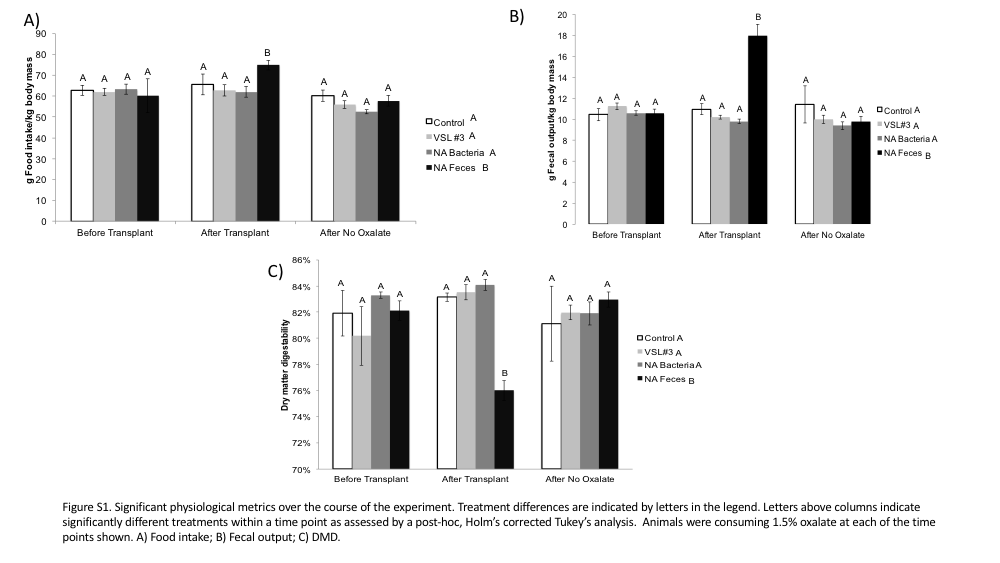

Supplement: FIG S1 [file sys005172139sf1.tif]

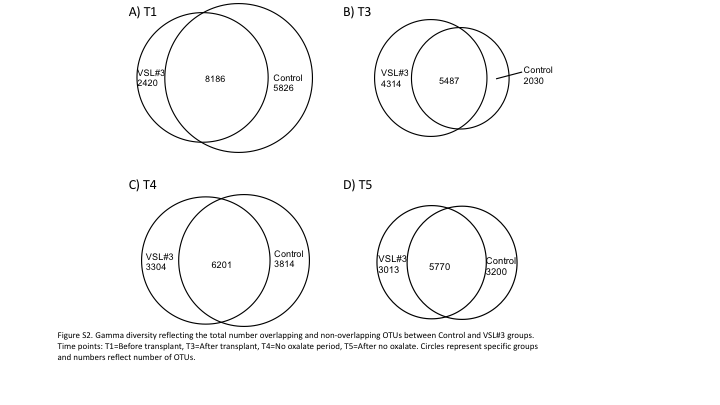

Supplement: FIG S2 [file sys005172139sf2.tif]

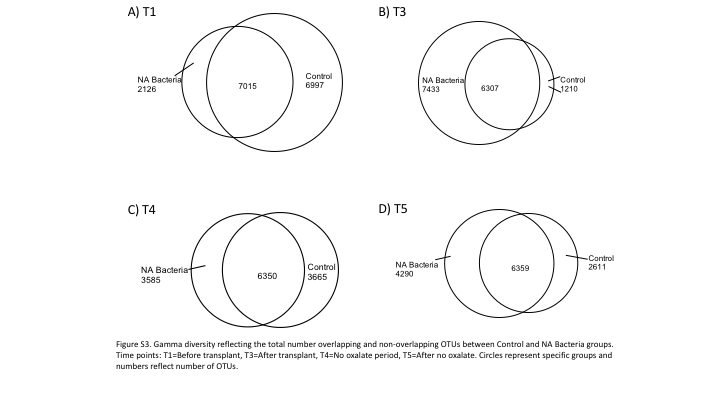

Supplement: FIG S3 [file sys005172139sf3.tif]
