# Supplementary material for: The Induction of Oxalate Metabolism In Vivo Is More Effective with Functional Microbial Communities than with Functional Microbial Species
Source: mSystems. 2017 Sep 26;2(5):e00088-17. doi: 10.1128/mSystems.00088-17 (PMC5613171; doi:10.1128/mSystems.00088-17)
Supplement: TABLE S1 [file sys005172139st4.pdf]

Lowest Assigned Taxonomy	Taxonomic Level	No. of OTUs	*r*	*FDR*
Ralstonia	genus	1	0.91	<0.001
Burkholderiales	order	2	0.61 +/- 0.184	<0.001
Oxalobacter formigenes	species	6	0.61 +/- 0.051	<0.001
Oxalobacteraceae	family	25	0.58 +/- 0.069	<0.001-0.006
Blautia producta	species	1	0.49	<0.001
Allobaculum	genus	4	0.48 +/- 0.015	<0.001
Sutterella	genus	2	0.45 +/- 0.016	<0.001-0.019
ML615J-28	order	17	0.43 +/- 0.016	<0.001-0.009
Roseburia	genus	2	0.42 +/- 0.19	<0.001-0.013
Odoribacter	genus	11	0.42 +/- 0.019	<0.001-0.01
RF39	order	6	0.42 +/- 0.019	0.006-0.043
Rikenellaceae	family	25	0.42 +/- 0.008	<0.001-0.045
Lachnospira	genus	2	0.41 +/- 0.08	<0.001-0.005
Elusimicrobiaceae	family	6	0.41 +/- 0.046	<0.001-0.048
S24-7	family	940	0.39 +/- 0.003	<0.001-0.049
Clostridium	genus	5	0.38 +/- 0.042	<0.001-0.032
Turicibacter	genus	19	0.38 +/- 0.015	<0.001-0.02
Unassigned	NA	236	0.38 +/- 0.005	<0.001-0.049
Clostridiales	order	356	0.38 +/- 0.004	<0.001-0.05
Ruminococcaceae	family	128	0.38 +/- 0.007	<0.001-0.05
Blautia	genus	1	0.38	<0.001
Butyrivibrio	genus	1	0.38	<0.001
Mogibacteriaceae	family	1	0.38	<0.001
Bacilli	class	5	0.37 +/- 0.046	<0.001-0.04
Ruminococcus gnavus	species	7	0.37 +/- 0.014	<0.001-0.003
Clostridiaceae	family	2	0.37 +/- 0.009	<0.001-0.001
YS2	order	62	0.37 +/- 0.009	<0.001-0.049
Lachnospiraceae	family	96	0.37 +/- 0.007	<0.001-0.049
Acidobacteriaceae	family	1	0.36	0.001
Adlercreutzia	genus	1	0.36	0.001
Desulfotomaculum	genus	1	0.36	0.001
Streptococcaceae	family	1	0.36	0.001
Veillonella dispar	species	1	0.36	0.001
Erysipelotrichaceae	family	2	0.35 +/- 0.037	<0.001-0.01
Enterococcus	genus	3	0.35 +/- 0.026	<0.001-0.006
Bacteroidales	order	4	0.35 +/- 0.019	<0.001-0.007
Bacteroides caccae	species	7	0.35 +/- 0.019	<0.001-0.016
Oscillospira	genus	42	0.35 +/- 0.017	<0.001-0.047
Desulfovibrio	genus	21	0.35 +/- 0.016	<0.001-0.042
Ruminococcus	genus	30	0.35 +/- 0.014	<0.001-0.045
Lactobacillus reuteri	species	3	0.35 +/- 0.012	<0.001-0.005
Bacteroides	genus	23	0.35 +/- 0.01	<0.001-0.027
Ruminococcus flavefaciens	species	33	0.35 +/- 0.01	<0.001-0.049
Lactobacillus	genus	52	0.35 +/- 0.007	<0.001-0.038
Granulicatella	genus	1	0.35	0.002
Bacillales	order	3	0.34 +/- 0.054	<0.001-0.03
Bifidobacterium	genus	8	0.34 +/- 0.024	<0.001-0.034
Faecalibacterium prausnitzii	species	2	0.34 +/- 0.017	0.001-0.005
Coprococcus	genus	13	0.34 +/- 0.012	<0.001-0.026
Parabacteroides	genus	4	0.33 +/- 0.036	<0.001-0.045
Coriobacteriaceae	family	4	0.33 +/- 0.02	0.001-0.034
RF32	order	3	0.33 +/- 0.001	0.005
Anaerostipes	genus	1	0.33	0.004
Oxalobacter	genus	1	0.33	0.005
Lactobacillaceae	family	3	0.32 +/- 0.022	0.001-0.019
Streptococcus	genus	15	0.32 +/- 0.006	0.001-0.034
Barnesiellaceae	family	1	0.32	0.008
Collinsella aerofaciens	species	4	0.31 +/- 0.075	0.006-0.019
Bilophila	genus	6	0.31 +/- 0.02	<0.001-0.05
Lactobacillales	order	2	0.31 +/- 0.016	0.006-0.02
Actinomyces	genus	2	0.30 +/- 0.03	0.003-.039
Bifidobacteriaceae	family	1	0.30 +/- 0.03	0.014
Parabacteroides distasonis	species	1	0.30	0.013
Akkermansia	genus	2	0.29 +/- 0.03	0.006-0.05
Coprobacillus	genus	1	0.29 +/- 0.03	0.02
Desulfovibrionaceae	family	5	0.29 +/- 0.014	0.002-0.042
Enterococcaceae	family	1	0.29	0.014
Acinetobacter	genus	1	0.28	0.034
Bifidobacterium pseudolongum	species	1	0.28	0.034
Enterobacteriaceae	family	1	0.28	0.029
Pediococcus	genus	1	0.28	0.034
S24-7	family	4	-0.28 +/- 0.008	0.01-0.042

Table S1. Microbial OTUs (out of 32,927) exhibiting a positive significant Pearson's correlation with the relative abundance of *Oxalobacteraceae.*
