# Supplementary material for: The Induction of Oxalate Metabolism In Vivo Is More Effective with Functional Microbial Communities than with Functional Microbial Species
Source: mSystems. 2017 Sep 26;2(5):e00088-17. doi: 10.1128/mSystems.00088-17 (PMC5613171; doi:10.1128/mSystems.00088-17)
Supplement: TABLE S2 [file sys005172139st5.pdf]

Diet	Time-point	# of days	Purpose
0-0.05% oxalate	T1	5	Quantify endogenous oxalate excretion; acclimate microbiota to a no oxalate diet
1.5% oxalate	T2	3	Quantify oxalate degradation; acclimate microbiota to 1.5%
1.5% oxalate + microbial transplants	T3	3	Introduce microbial communities to recipients; quantify the change in oxalate degradation
0-0.05% oxalate	T4	7	Washout any empherally colonized oxalate-degrading bacteria
1.5% oxalate	T5	3	Quantify the persistence of the oxalate-degrading function and bacteria from transplants

Table S2. Timeline for the diet trial to quantify the effect of oxalate and microbial transplants on oxalate degradation. Feces were collected for microbial inventories at the end of each time point.
