# Supplementary material for: The Induction of Oxalate Metabolism In Vivo Is More Effective with Functional Microbial Communities than with Functional Microbial Species
Source: mSystems. 2017 Sep 26;2(5):e00088-17. doi: 10.1128/mSystems.00088-17 (PMC5613171; doi:10.1128/mSystems.00088-17)
Supplement: TABLE S3 [file sys005172139st6.pdf]

Ingredients	g/kg
casein	200.0
L-cystine	3.0
corn starch	300.5
maltodextrin	120.0
sucrose	100.0
soybean oil	70.0
cellulose	150.0
mineral mix	42.0
vitamin mix	12.0
choline bitartrate	2.5
TBHQ antioxidan	0.014
