# Supplementary material for: The Induction of Oxalate Metabolism In Vivo Is More Effective with Functional Microbial Communities than with Functional Microbial Species
Source: mSystems. 2017 Sep 26;2(5):e00088-17. doi: 10.1128/mSystems.00088-17 (PMC5613171; doi:10.1128/mSystems.00088-17)
Supplement: TABLE S4 [file sys005172139st7.pdf]

Group	Time point	No. of Samples
NALB	1	6
NALB	3	6
NALB	4	6
NALB	5	6
Control	1	6
Control	3	6
Control	4	6
Control	5	6
VSL #3	1	6
VSL #3	3	6
VSL #3	4	6
VSL #3	5	6
WR Bacteria	1	6
WR Bacteria	3	6
WR Bacteria	4	6
WR Bacteria	5	6
WR Feces	1	6
WR Feces	3	6
WR Feces	4	6
WR Feces	5	6
**Total**		120
